# Melatonin as a potential inhibitory agent in head and neck cancer

**DOI:** 10.18632/oncotarget.20079

**Published:** 2017-08-09

**Authors:** Chia-Ming Yeh, Shih-Chi Su, Chiao-Wen Lin, Wei-En Yang, Ming-Hsien Chien, Russel J. Reiter, Shun-Fa Yang

**Affiliations:** ^1^ Institute of Medicine, Chung Shan Medical University, Taichung, Taiwan; ^2^ Whole-Genome Research Core Laboratory of Human Diseases, Chang Gung Memorial Hospital, Keelung, Taiwan; ^3^ Institute of Oral Sciences, Chung Shan Medical University, Taichung, Taiwan; ^4^ Department of Dentistry, Chung Shan Medical University Hospital, Taichung, Taiwan; ^5^ Department of Medical Research, Chung Shan Medical University Hospital, Taichung, Taiwan; ^6^ Graduate Institute of Clinical Medicine, Taipei Medical University, Taipei, Taiwan; ^7^ Department of Cellular and Structural Biology, The University of Texas Health Science Center, San Antonio, TX, USA

**Keywords:** melatonin, head and neck cancers, metastasis, matrix metalloproteinase

## Abstract

Melatonin is a molecule secreted by the pineal gland; it is an important regulator of sleep and circadian rhythms. Through multiple interrelated mechanisms, melatonin exhibits various inhibitory properties at different stages of tumor progression. Many studies have explored the oncostatic effects of melatonin on hormone-dependent tumors. In this review, we highlight recent advances in understanding the effects of melatonin on the development of head and neck cancers, including molecular mechanisms identified through experimental and clinical observations. Because melatonin exerts a wide range of effects, melatonin may influence many mechanisms that influence the development of cancer. These include cell proliferation, apoptosis, angiogenesis, extracellular matrix remodeling through matrix metalloproteinases, and genetic polymorphism. Thus, the evidence discussed in this article will serve as a basis for basic and clinical research to promote the use of melatonin for understanding and controlling the development of head and neck cancers.

## INTRODUCTION

Head and neck cancers constitute the sixth most common malignancy in the world. Most head and neck cancers occur in the epithelial lining of the oral cavity, hypopharynx, larynx, and oropharynx. Squamous cell carcinoma is the most frequent type, accounting for approximately 90% of all head and neck cancers. Approximately 50% of all head and neck cancers occur in the oral cavity [1, 2]. Alcohol and tobacco use are major risk factors for most head and neck cancers; studies have revealed that the incidence of head and neck cancers is higher in regions with high rates of alcohol and tobacco consumption [3, 4]. Also, experimental and clinical data indicate that human papillomavirus infection is also related to the development of head and neck cancers [5, 6]. The overall 5-year survival for head and neck cancers is approximately 50%; this statistic has not changed much in the past few decades.

### Melatonin

**Figure 1:**
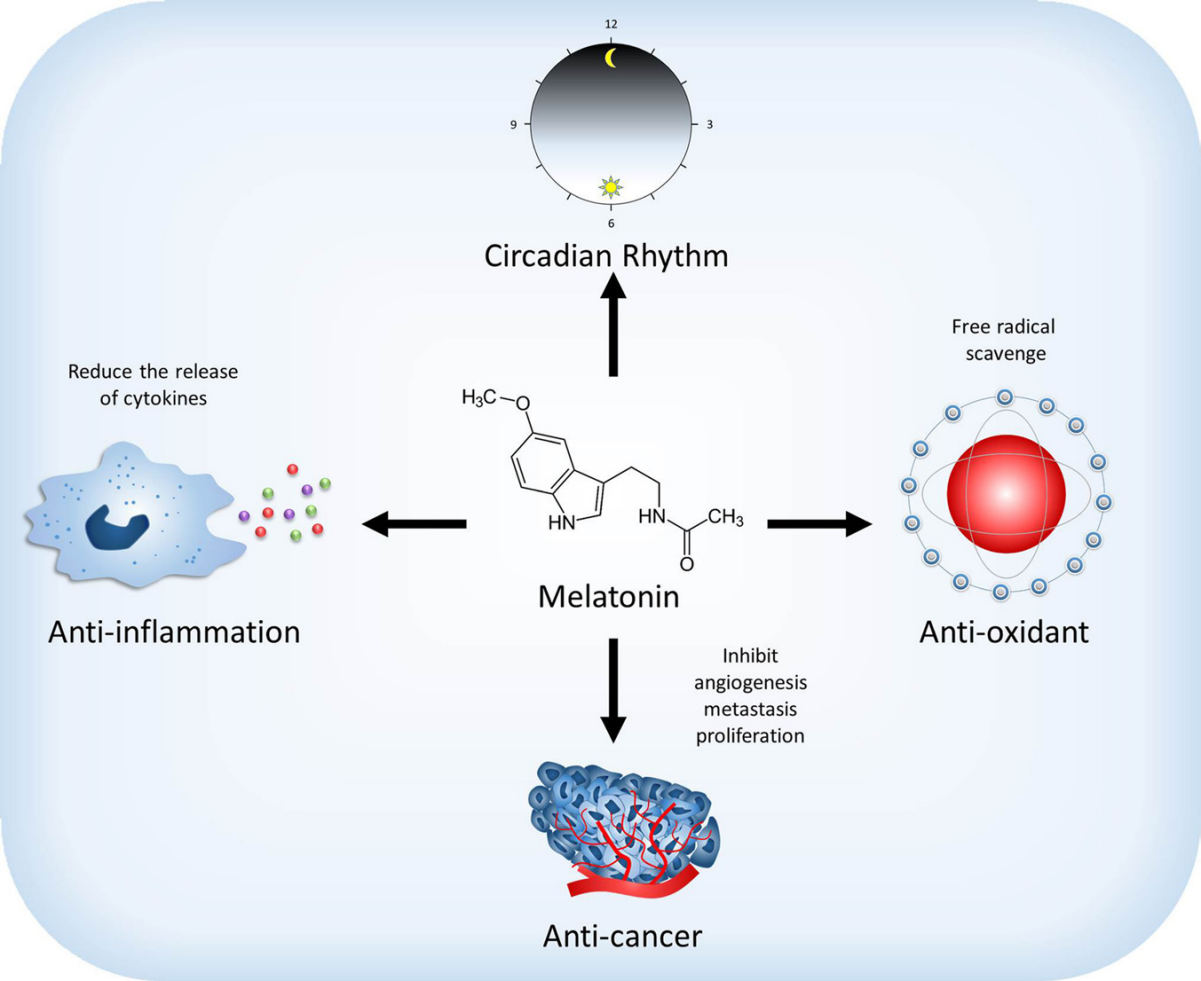
Effect of melatonin on the physiological and pathological functions Melatonin regulates sleep and circadian rhythms. Moreover, melatonin also has anti-oxidant and anti-inflammation abilities to scavenge free radical and reduce the release of cytokines. Melatonin may reduce the development of cancer through affecting the mechanism of angiogenesis, metastasis and proliferation.

Melatonin, chemically named N-acetyl-5-methoxytryptamine, was discovered in the bovine pineal gland by Lerner in 1958 [7]. Since its discovery, melatonin has been extensively studied, and numerous benefits have been reported (Figure 1). Melatonin is widely distributed in bacteria, unicellular organisms, algae, plants, invertebrates, and in many organs of vertebrates [8–11]. Its production has also been documented in nonvertebrates and plants that lack a pineal gland [8, 12]. In mammals, melatonin is a derivative of tryptophan. It is synthesized in the pineal gland and rhythmically secreted into the blood [13–15] and into the cerebrospinal fluid (CSF) [16, 17]. The production of melatonin begins at night during darkness; in humans, melatonin reaches maximal concentration near the middle of the dark period [13].

Melatonin is a molecule with pleiotropic functions; it is involved in regulating the circadian rhythms of physiological functions, including blood pressure, seasonal reproduction, and sleep timing [18–21]. Various receptor subtypes are available for melatonin binding and activation. Studies have demonstrated that membrane melatonin receptors are on perhaps all cells including retina, brain, suprachiasmatic nucleus, pituitary gland, ovary, cerebral artery, peripheral artery, kidney, pancreas, fat, and immune cells [22–24]. Among several signaling mechanisms, melatonin actions are activated through high-affinity G protein–coupled receptors (GPCRs), including the MT1 and MT2 [25–27]; they have amino acid sequences that have 60% homology and different chromosomal localization [28, 29]. These melatonin receptors have potential glycosylation sites at their N-terminus regions and have casein kinase 1 and 2, protein kinase A, and protein kinase C phosphorylation sites, which may be involved in the regulation of receptor function, as evidenced in other GPCRs [30]. In addition to the well described membrane melatonin receptors, melatonin has binding sites in the nucleus [31, 32] and couples with calmodulin and quinone reductase 2 in the cytosol [33, 34]. Major receptor-mediated actions of melatonin include the regulation of circadian rhythms including sleep [19, 35] as well as other actions such as anti-cancer and anti-inflammatory actions [36–47].

In normal cells, melatonin and its derivatives are powerful free radical scavengers and multi-faceted antioxidants [11, 48, 49]. Melatonin executes its direct free radical scavenging actions via non-receptor-mediated mechanisms. Melatonin reduces oxidative stress that is associated with many diseases, including optic neuritis, myocardial ischemia and neurological disease [50–53]. Compared with other antioxidants, melatonin has an equal or superior ability to protect tissues from oxidative injury even when compared with synthetic mitochondria-targeted antioxidants [54]. One difference between melatonin and other free radical scavengers is its amphiphilicity, which allows melatonin to distribute throughout the subcellular environment although in differing concentrations among organelles [55]. Furthermore, melatonin is estimated to detoxify up to 10 free radicals through the AFMK pathway, thus increasing its effective concentration [56]. As noted above, melatonin also interacts with the detoxifying enzyme, quinone reductase 2 [34]. Although the mechanism of this interaction is unclear, this coupling may be related to the regulation of cell redox status [57]. Also, the interaction between melatonin and calmodulin may be involved in antioxidant signaling and other signaling processes that reduce the oxidative burden [33]. In addition to directly scavenging toxic oxygen-based reactants, melatonin also indirectly limits oxidative damage by stimulating a variety of antioxidant enzymes which remove free radicals before they damage essential molecules [58–61].

Oxidative stress is defined as the imbalance between an organism's cellular production of oxidative agents, such as reactive oxygen species (ROS), and the organism's antioxidant capacity, ROS participate in many different cellular processes during physiological and pathologic reactions [62].

In cancer cells, melatonin functions as a conditional pro-oxidant [63–65]. For example, Wolfler et al. [65] showed that melatonin stimulates ROS generation and causes Fas-induced apoptosis in human leukemic cells. Osseni et al. [66] also reported that melatonin can be both anti-oxidant and pro-oxidant in a human HepG2 liver cell line. In cervical cancer cells, Pariente et al. [67] reported that melatonin enhances cisplatin-induced cytotoxicity and apoptosis due to ROS overproduction. Moreover, Um et al. [68] demonstrated that melatonin attenuates oxaliplatin-induced apoptosis and anti-oxidant action in renal carcinoma Caki cells.

ROS production promotes the release of inflammatory mediators, including the activation of redox-regulated transcription factors, such as NF-κB, to produce cytokines by activating the intracellular inflammatory signaling pathways [69]. Melatonin scavenges a variety free radicals in body fluids, cells and *in vitro* [70–72]. These effects enable melatonin to reduce the level of ROS and decrease oxidative pathologies such as seen in atherosclerosis, neurodegenerative diseases, hypertension, ischemia, and cancer [73–75].

Clinical evidence that has accumulated in the last two decades suggests that melatonin inhibits the growth of many cancers, including cervical cancer, ovarian cancer, breast cancer, and colon cancer [76–79]. The drop in melatonin that occurs during aging correlates with immunosenescence, neurodegenerative disorders, and cancer. Moreover, breast cancer cell proliferation is higher during the daytime (when melatonin levels are low) than during the night (when melatonin levels are high). Thus, the age-associated reduction of melatonin with advancing age may promote the proliferation of breast cancer as well as other cancer types [80].

Melatonin released into the oral cavity by saliva may have protective effects on many oral disorders, such as herpes viral infections and *Candida* infection, periodontal diseases, xerostomia, local inflammatory processes, oral ulcers, and oral cancer [81, 82]. Moreover, Ortiz et al. [83] reported that melatonin gel applied in the oral cavity can reduce the development of erythema and prevents ulcer formation; therefore, it is a potential preventive therapy for radiotherapy-induced oral mucositis. Of melatonin's various effects on oral disorders, the most widely studied is its role in periodontal disease, which is related to the anti-inflammatory and antioxidant properties of melatonin. In addition, radical scavengers, such as melatonin, reduce oxidative damage in *in vivo* and *in vitro* in head and neck cancers [84].

**Figure 2:**
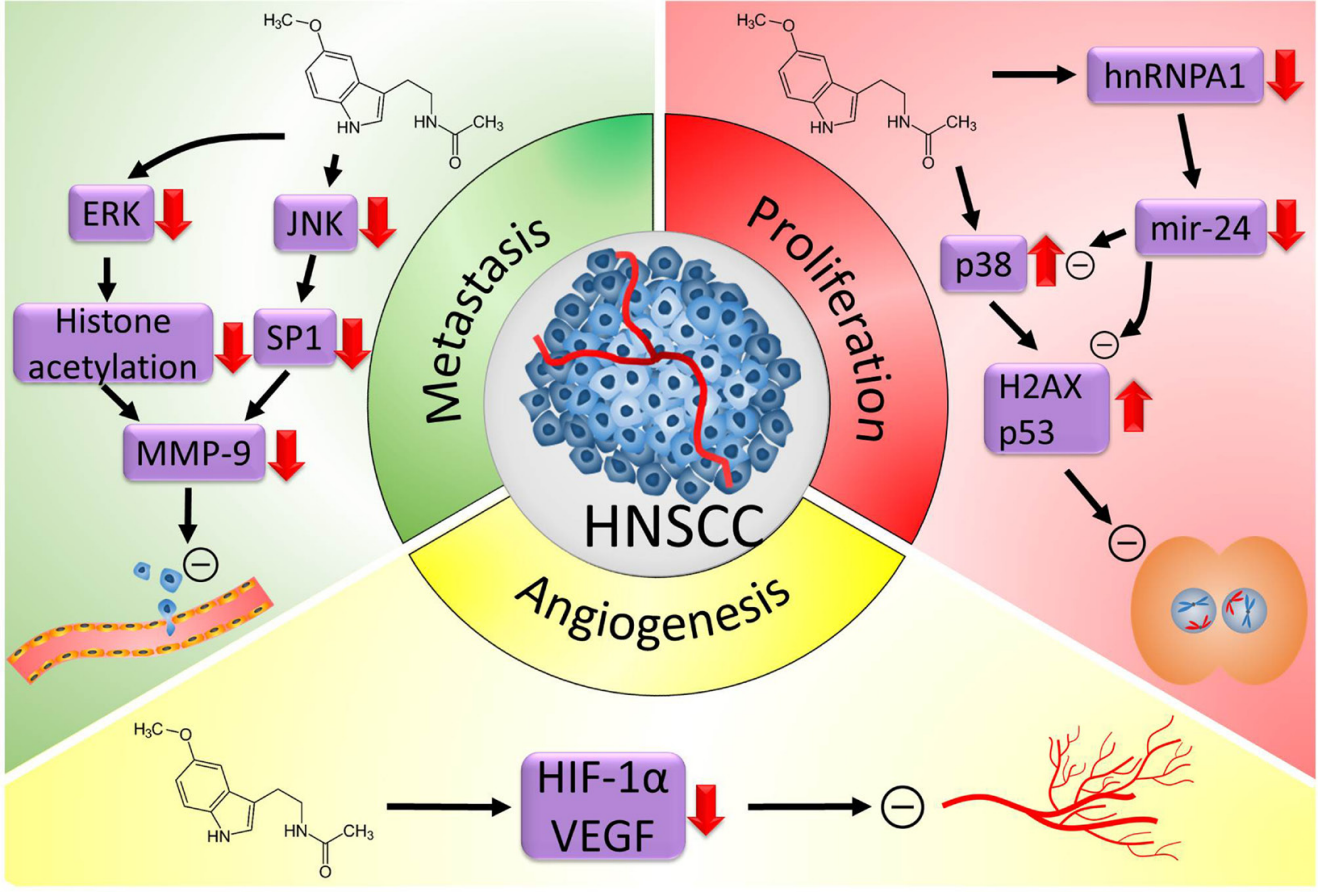
Proposed oncostatic actions of melatonin on head and neck cancer (HNSCC) Melatonin treatment reduces HNSCC cell metastasis through inhibiting the expression of MMP-9 by targeting the ERK/JNK signal pathway to mediate histone acetylation and SP-1 expression. Melatonin inhibits HNSCC cell proliferation through upregulating p38, H2AX and p53 expression and downregulating the expression of hnRNPA1 and mir-24. Melatonin supplementation suppresses NHSCC cells angiogenesis by reducing the expression of angiogenesis molecular markers, HIF-1α and VEGF. ERK, mitogen-activated protein kinase; JNK, c-Jun N-terminal kinase; MMP-9, matrix metalloproteinase 9; H2AX, H2A histone family member X; hnRNPA1, heterogeneous nuclear ribonucleoprotein A1; HIF-1α, hypoxia inducible factor 1 alpha subunit; VEGF, vascular endothelial growth factor.

This article focuses on the role of melatonin in the treatment of head and neck cancers (Figure 2). Since head and neck cancers are among the list of life-threatening diseases with poor survival rates, an agent that retards or controls the occurrence of these tumors is in need of identification. Therefore, we focus the effects of melatonin on the development of head and neck cancers, including molecular mechanisms identified through experimental and clinical observations (Table 1).

**Table 1:** Melatonin and head and neck cancer

Research object	Measures	Outcome	References
250 metastatic solid tumour patients including 104 lung cancers, 77 breast cancers, 42 gastrointestinal tract neoplasms, 27 head and neck cancers	The percentage of 1-year survival was calculated in metastatic solid tumour patients that were randomised to be treated with chemotherapy alone or chemotherapy plus melatonin	The 1-year survival rate and the objective tumour regression rate in patients concomitantly treated with MLT were significantly higher than in those who received chemotherapy (CT) alone	Lissoni P, et al. [101]
Oral squamous cell carcinoma cell lines, SCC-9 and SCC-25	*In vitro* studies of oral squamous cell carcinoma cell lines treated with melatonin	Melatonin inhibits expression of molecular markers of angiogenesis, VEGF and HIF-1 in SCC-9 cell line.	Goncalves Ndo N, et al. [140]
Oral squamous cell carcinoma cell lines, HSC-3 and OECM-1	*In vitro* studies of oral squamous cell carcinoma cell lines treated with melatonin	Melatonin affect the motility of HSC-3 and OECM-1 cells *in vitro* throughtargeting the ERK pathways to mediate histone acetylation and then inhibit MMP-9 transcription	Yeh CM, et al. [131]
Nasopharyngeal carcinoma cell lines, HONE- 1, NPC- 39, and NPC- BM	*In vitro* studies of nasopharyngeal carcinoma cell lines treated with melatonin	Melatonin suppresses the motility of nasopharyngeal carcinoma cell lines *in vitro* via inhibiting SP- 1- DNA binding ability to regulate MMP- 9 gene expression	Ho HY, et al. [132]
618 patients with oral cancer and 560 non-cancer controls	MTNR1A polymorphism was measured in genomic DNA samples extracted from blood samples	Oral cancer patients with the T/T allele of MTNR1A gene variants with betel nut chewing habit have a high correlation to develop a higher risk for late clinical staging and lymph node metastasis	Lin FY, et al. [163]

### Melatonin: antiproliferative and pro-apoptotic actions in head and neck cancer

The difference between head and neck cancer cells and normal cells is that head and neck cancer cells exhibit uncontrolled and sustainable growth. Normal cells regulate growth, division, and the cell cycle through complex cell growth messages (growth-promoting signals) that maintain a constant number and size of cells. Most head and neck cancer cells are over-reliant on specific signaling pathways (signaling transduction pathways) which promote cancer cell proliferation [85]. Aberrations in the critical pathways that regulate cell survival and cell proliferation are necessary for establishing all tumors [86]. The deregulation of cell proliferation and inhibition of apoptosis are common mechanisms involved in the development of all cancers [38, 87, 88]. Therefore, identifying the difference between tumor cells and normal cells and the appropriate use of this information are key issues in cancer treatment [89].

Empirical evidence unequivocally documents that toxic oxygen derivatives influence the balance between cell proliferation and apoptosis. If the mechanisms of apoptosis are overwhelmed, cell proliferation may predominate leading to tumor formation. Thus, radical scavengers such as melatonin may regulate the proliferation of head and neck cancers through the reduction of oxidative damage [84]. Multiple proliferative signals affect apoptosis programming by inducing Alternative reading frame protein (ARF), an alternate product of the INK4a locus, one of its functions is to trigger the upregulation of p53 by inhibiting MDM-2 [90, 91].

Apoptosis, a type of programmed cell death, is an important mechanism that normally occurs in all tissues. This mechanism is important for ridding tissues from damages on diseased cells. Apoptosis often also occurs during aging and development and acts as a homeostatic mechanism for maintaining the stability of the cell population in the tissues. It is also a defense mechanism for the immune response and for cells damaged by noxious agents or disease [92]. Although various physiological and pathological stimuli or conditions can trigger apoptosis, not all cells die from the same stimulus. Drugs or radiation for cancer chemotherapy also cause DNA damage in some cells, which triggers apoptosis through a p53-dependent pathway [93, 94]. Cell apoptotic programs involve either the intrinsic or extrinsic pathway, as distinguished by the source of the death signal and initiator caspases involved [95]. The intrinsic apoptosis is a response to internal damage (e.g., mitochondrial stress or chromosomal defects) with the activation of caspase 9, whereas the extrinsic apoptotic pathway is mediated through membrane-bound death receptors (e.g., FAS or tumor necrosis factor receptors) triggered by external stimuli, which activate caspase 8.

The tumor suppressor gene *TP53*, which encodes p53, is central to DNA damage recognition, DNA repair, cell cycle regulation, proliferation, and apoptosis [96]. Conceivably, multiple p53-related pathways play fundamental roles in the development of cancer, which explains why this gene is most commonly mutated in human malignancies [97]. Substantial evidence has indicated that melatonin regulates proliferation and apoptosis in various cancer types through the p53 signaling pathway, revealing a mechanistic link between melatonin and p53 signaling. Of note, melatonin inhibits cell proliferation by arresting the cell cycle through a p53-mediated rise in the expression of p21WAF1 protein in breast cancer [98].

Melatonin treatment also inhibits the proliferation of hepatocarcinoma cells by promoting cell apoptosis via the upregulation of mitogen-activated protein kinase family members, p38 and c-Jun N-terminal kinase (JNK)-1, -2, and -3, as well as the elevation of caspase-8 activity [99]. Additionally, melatonin affects the expression of miR-24 microRNA, whose downstream target genes modulate p38 and p53, in colon cancer, breast cancer, and head and neck cancers [100]. A clinical study further revealed that the 1-year survival rate and objective tumor regression rate were significantly higher in cancer patients who were concomitantly treated with melatonin than in those receiving chemotherapy alone [101]. These findings suggest that melatonin contributes to suppression of cell proliferation and induction of apoptosis in head and neck cancer.

### Melatonin counteracts metastasis in head and neck cancer

Cancer metastasis, the spread of cancer cells from the tissues or organs of tumor origin to other sites, is the leading cause of death in cancer patients [39, 102–109]. The cascade of metastasis can be divided into three processes: invasion, intravasation, and extravasation [39, 102, 110, 111]. In the process of invasion, the loss of cell-cell adhesion allows malignant tumor cells to escape from primary tumors and to invade the surrounding matrix [112]. This process involves the secretion of enzymes such as matrix metalloproteinases (MMPs) that degrade the extracellular matrix (ECM) and basement membrane [113] and the expression/suppression of proteins involved in controlling cell motility and migration [114]. MMPs, belong to a family of enzymes that contain zinc atoms at their active sites, are secreted by inflammatory phagocytes, connective tissue cells, and many different transformed cells [115]. As a key regulator of ECM remodeling, MMPs function to break down most of the ECM components, including elastin, laminin, collagen, serpin, and fibronectin [115, 116]. Among MMPs, MMP-2, MMP-9 and membrane-type matrix metalloproteinases (MT-MMPs) are believed to play an important role in cancer invasion and metastasis [109, 117–122]. Mounting evidence has indicated that the inhibition of MMP-2 and MMP-9 activity reduces cancer cell metastasis in head and neck cancer [123–127]. Moreover, in highly metastatic head and neck tumors, MMP-9 is overexpressed compared with that in normal tissues [128–130]. Previous studies also mentioned that melatonin inhibited the gene expression of MMP-9 in head and neck cancers [131, 132]. The study of melatonin-regulated head and neck cancer metastases has demonstrated that melatonin targeted the ERK/JNK pathways to reduce MMP-9 transcription and cancer cell invasion through modulating histone acetylation and SP1 activation [131].

In addition to ECM remodeling, tumor progression requires the activation of angiogenesis, a process defined as the formation of new blood vessels from pre-existing structures. Blood vessels surrounding the tumor not only supply the oxygen and nutrients [133] but also permit the invasion of cancer cells into the circulatory system and their migration to distal sites [134, 135]. The process of tumor angiogenesis is orchestrated by multiple signaling pathways elicited by interactions between the tumor cells and the surrounding stroma. Various proteins have been demonstrated to be pro-angiogenic, including but not limited to angiogenin, basic fibroblast growth factor, epidermal growth factor, granulocyte colony-stimulating factor, hepatocyte growth factor, interleukin-8, placental growth factor, platelet-derived endothelial growth factor, transforming growth factor (TGF)-α, TGF-β, tumor necrosis factor-α, and vascular endothelial growth factor (VEGF). Among them, the VEGF family and its receptors have drawn considerable attention to the field of tumor angiogenesis [136, 137].

Recently, multiple reports have shown that melatonin can decrease the expression of VEGF in various cancers [138, 139]. In oral carcinoma and oral cancer cell lines, melatonin inhibited the expression of the proangiogenic factors, HIF1α and VEGF revealing an effect of melatonin on inhibition of angiogenic responses in oral cancer [140]. These results suggest that melatonin has the potential to inhibit the invasion and metastasis of head and neck cancer by modulating tumor angiogenesis.

### Polymorphisms of the melatonin receptor genes MTNR1A and MTNR1B in head and neck cancer

With the rapid development of the Human Genome Project, the science of pharmacogenetics, which incorporates information on the genetic variability for predicting the response to treatment, is booming. Because the therapeutic index of many chemotherapy drugs for cancer is narrow, a better understanding of pharmacogenetics on cancer chemotherapy may offer individualized cancer treatment [141]. Studies have shown that the variations of individual genomes and tumor genomes affect the drug response in tumors and the risk of developing cancers [142, 143].

Single-nucleotide polymorphism (SNP) occurs as a variation in one nucleotide, which occurs at specific locations in the genome, and each variation has an appreciable degree of detectability in the population [144]. The systematic analysis of candidate gene association has revealed that SNPs in genes involved in cell cycle control ECM remodeling, DNA repair, folate metabolism, and that carcinogen metabolism may be associated with increased susceptibility to cancers [145–153].

Mounting evidence indicates that melatonin exhibits oncostatic properties in many cancer types mainly mediated by its membrane-bound receptors, melatonin receptor 1A (encoded by *MTNR1A*) and 1B (*MTNR1B*). Increased expressions of *MTNR1A* and *MTNR1B* have been shown to promote the inhibitory actions of melatonin on the growth of cancer cells [154, 155]. It is documented that the variations of melatonin receptor genes are associated with susceptibility to many diseases [156–160].

The frequencies of the genotypes and allelotypes of SNP rs2119882 for the *MTNR1A* gene significantly differ between patients with polycystic ovary syndrome and healthy controls [157], while another SNP in the *MTNR1A* gene, rs7665392, may contribute to breast cancer susceptibility [161]. In addition to *MTNR1A*, associations of *MTNR1B* rs3781638 and rs10765576 with osteoporosis [162] and breast cancer [161], respectively, have been reported. Melatonin receptor gene polymorphisms in combination with environmental parameters have been correlated with the risk for oral cancer [163]. Oral cancer patients who habitually chewed betel nut and carried the T/T allele of *MTNR1A* rs13140012, were more prone to develop lymph node metastasis and late-stage tumors.

### Summary and concluding remarks

Melatonin is not only a significant immunomodulatory compound but is also a powerful antioxidant that can effectively protect critical molecules from ROS-mediated damage, thereby serving as a vital regulator in cancer suppression. Moreover, melatonin has a very low toxicity profile and is not associated with significant side effects; hence, melatonin has been safely used in various clinical settings [164–166]. In this article, we discussed that melatonin regulates cell proliferation, apoptosis, and metastasis, to exert an anticancer effect. These data provide clues for further clarifying the mechanisms underlying melatonin-mediated inhibition of tumor progression as well as for designing clinical trials for combinational therapies against head and neck cancer.

## Abbreviations

ECM: extracellular matrix
GPCRs: G protein-coupled receptors
JNK: c-Jun N-terminal kinase
MMP: matrix metalloproteinase
MT-MMPs: membrane-type matrix metalloproteinases
MTNR1A: melatonin receptor 1A
MTNR1B: melatonin receptor 1B
ROS: reactive oxygen species
SNP: Single-nucleotide polymorphism
TGF: transforming growth factor
VEGF: vascular endothelial growth factor
